# Using Deep Learning to Identify High-Risk Patients with Heart Failure with Reduced Ejection Fraction

**DOI:** 10.36469/jheor.2021.25753

**Published:** 2021-07-29

**Authors:** Zhibo Wang, Xin Chen, Xi Tan, Lingfeng Yang, Kartik Kannapur, Justin L. Vincent, Garin N. Kessler, Boshu Ru, Mei Yang

**Affiliations:** 1 Merck & Co., Inc., Kenilworth, NJ, USA; College of Engineering and Computer Science, University of Central Florida, Orlando, FL, USA; 2 Merck & Co., Inc., Kenilworth, NJ, USA; 3 Amazon Web Services Inc., Seattle, WA, USA; 4 Amazon Web Services Inc., Seattle, WA, USA; Georgetown University, Seattle, WA, USA

**Keywords:** machine learning, heart failure, readmissions, worsening events, hospitalizations, deep learning

## Abstract

**Background:** Deep Learning (DL) has not been well-established as a method to identify high-risk patients among patients with heart failure (HF).

**Objectives:** This study aimed to use DL models to predict hospitalizations, worsening HF events, and 30-day and 90-day readmissions in patients with heart failure with reduced ejection fraction (HFrEF).

**Methods:** We analyzed the data of adult HFrEF patients from the IBM® MarketScan® Commercial and Medicare Supplement databases between January 1, 2015 and December 31, 2017. A sequential model architecture based on bi-directional long short-term memory (Bi-LSTM) layers was utilized. For DL models to predict HF hospitalizations and worsening HF events, we utilized two study designs: with and without a buffer window. For comparison, we also tested multiple traditional machine learning models including logistic regression, random forest, and eXtreme Gradient Boosting (XGBoost). Model performance was assessed by area under the curve (AUC) values, precision, and recall on an independent testing dataset.

**Results:** A total of 47 498 HFrEF patients were included; 9427 with at least one HF hospitalization. The best AUCs of DL models without a buffer window in predicting HF hospitalizations and worsening HF events in the total patient cohort were 0.977 and 0.972; with a 7-day buffer window the best AUCs were 0.573 and 0.608, respectively. The best AUCs in predicting 30- and 90-day readmissions in all adult patients were 0.597 and 0.614, respectively. An AUC of 0.861 was attained for prediction of 90-day readmission in patients aged 18-64. For all outcomes assessed, the DL approach outperformed traditional machine learning models.

**Discussion:** The DL approach can automate feature engineering during the model learning, which can increase the clinical applicability and lead to comparable or better model performance. However, the lack of granular clinical data, and sample size and imbalance issues may have limited the model’s performance.

**Conclusions:** A DL approach using Bi-LSTM was shown to be a feasible and useful tool to predict HF-related outcomes. This study can help inform the future development and deployment of predictive tools to identify high-risk HFrEF patients and ultimately facilitate targeted interventions in clinical practice.

## INTRODUCTION

Heart failure (HF) is the second most expensive medical condition in terms of health-care spending per individual, with costs higher than those related to treating severe cancers.^1^ Given the substantial economic burden associated with HF and the complex nature of its management, it is important for providers and payers to proactively identify high-risk patients and provide optimal treatment in a timely manner. However, the creation and utilization of prognostic tools and the identification of risk factors in clinical practice remain in an early phase, and models that predict and identify HF-related adverse outcomes (eg, hospitalizations and readmissions) have substantial room for improvement. Reviews of the predictive models for HF-related outcomes found that the accuracy of predicting HF hospitalization is significantly worse than for predicting mortality.^2^ Additionally, most of the existing predictive models have limitations that may impact prediction validity and reliability, such as including only a few pre-specified potential predictors, having a relatively small sample size, and being developed in a single clinical setting without external validation.^3^

Machine learning (ML) has been applied to the detection of HF, HF subtype and severity classification, and prediction of HF prognosis and outcomes.^3–6^ However, traditional ML models often need dedicated and expert-driven feature engineering and data pre-processing to achieve good performance on heterogeneous sources of clinical data. The process can be time-consuming and challenging to reuse across different disease/data source settings that are present in day-to-day clinical practice. Blindly dumping data into one-size-fits-all feature engineering pipelines with traditional ML models could lead to unsatisfactory model performance.^3^ Recently, deep learning (DL),^7^ a subset of ML, has been broadly applied to research areas of computer vision, voice recognition, and natural language processing and has several advantages over traditional ML models. First, DL methods can automatically extract patterns from large-scale, high-dimensional, structured, semi-structured, and unstructured data (eg, medical claims datasets, electronic health records, physician notes, and images). Second, deep neural networks can extract complex spatiotemporal feature representations, which can help identify hidden patterns and highly predictive variables that may be missed in traditional, hand-crafted feature engineering methods. This avoids heavy feature engineering tasks and preserves most of the inherent information in longitudinal and sequential data. In health care, DL models have also been used for predicting disease onset, prognosis, and health outcomes.^8–10^ For example, Rajkomar and colleagues (2018) demonstrated that the long short-term memory (LSTM) DL algorithm was superior to traditional non-DL predictive models for predicting health-care outcomes in the general inpatient population.^11^ In the area of HF, LSTM has been utilized to predict disease onset with promising performance.^12–14^ However, DL has not been well studied in predicting disease prognosis or outcomes such as hospitalization or worsening events among patients with HF.

The present study focused on patients with heart failure with reduced ejection fraction (HFrEF) and aimed to build multiple DL models to predict four types of outcomes: HF hospitalizations, worsening HF events, 30-day readmission following HF hospitalization, and 90-day readmission following HF hospitalization. The results were compared with non-DL models as well. The approach established by this study has the potential to help identify high-risk HF patients, which will improve the management of HF in real-world practice and may also identify patients in need of targeted interventions.

## MATERIALS AND METHODS

### Data Source

We analyzed data from the IBM® MarketScan® Commercial and Medicare Supplement Databases between January 1, 2015 and December 31, 2017. The databases contain medical and pharmacy claims data from a variety of employers and health plans across the United States. They include patient-level demographic data, medical and pharmacy claims, and enrollment information.

### Study Population

The general HFrEF population in this study comprised patients ≥18 years old with a confirmed diagnosis of HFrEF between January 1, 2016 and December 31, 2016. The date of the first claim with an HFrEF diagnosis in 2016 was defined as the index date. The diagnosis of HFrEF was considered confirmed if at least one of the following criteria was met: a) one inpatient claim with an HFrEF diagnosis using the International Classification of Diseases, Tenth Revision (ICD-10) codes I50.2x, I50.4x, or I50.1; b) two outpatient claims for HFrEF; or c) one outpatient claim for HFrEF (I50.2x, I50.4x, or I50.1) plus one outpatient claim with any HF diagnosis (I50.1, I50.2x, I50.3x, I50.4x, I50.8x, I50.9, or I11.0) on two different dates within 12 months.^15,16^ In addition, patients in our study population had to be continuously enrolled in a health plan for at least 12 months before and after the index date, with an allowed gap of fewer than 45 days between periods of continuous enrollment. Patients with evidence of heart transplant, a left ventricular assist device, adult congenital heart disease (eg, single ventricle disease), or amyloidosis prior to the index date were excluded.

### Study Design and Outcomes

The outcomes of interest were HF hospitalizations, worsening HF events, 30-day readmission following HF hospitalization, and 90-day readmission following HF hospitalization. HF hospitalization (yes/no) was defined as any inpatient claim after the index date with a diagnosis of HF using ICD-10 codes of I50.1, I50.2x, I50.3x, I50.4x, I50.8x, I50.9, and I11.0. Worsening HF events (yes/no) were defined as HF-related hospitalizations or outpatient intravenous diuretic use after the index date. The 30-day/90-day hospital readmission following HF hospitalization (yes/no) was defined as the first claim for a subsequent hospitalization within 30 or 90 days following the discharge date of the index HF hospitalization, which was the first HF hospitalization after the index date.

**Figure 1.**
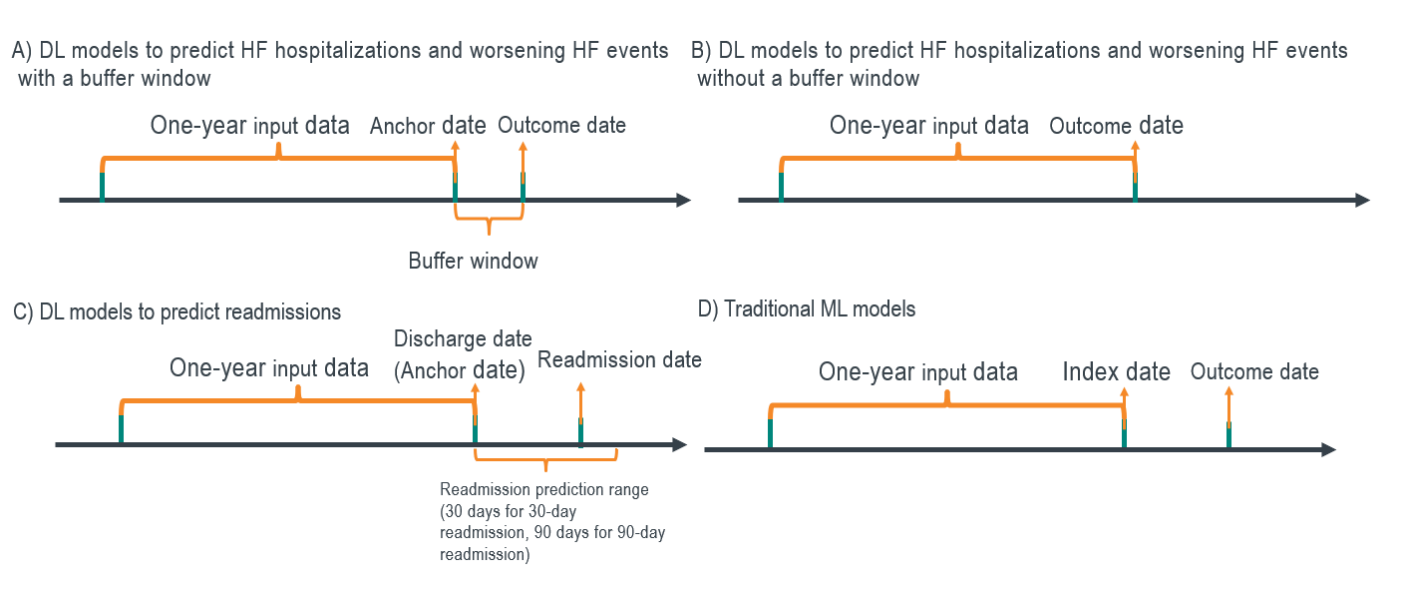
Study Design Abbreviations: DL, deep learning; HF, heart failure; ML, machine learning.

For DL models to predict HF hospitalizations and worsening HF events, we utilized two study designs: with and without a buffer window (Figure 1). First, for the traditional design without a buffer window, the event date was considered the outcome date for positive cases. For negative cases, a pseudo-outcome date was generated based on the distribution of the time gap between the index date and outcome date in the positive samples. This approach prevents the model from learning the difference between high- and low-risk groups from data artifacts caused by subjectively cutting the time window for the negative class based on the index date, a certain number of days post-index, or the last of any activity dates. Second, we also utilized a new study design incorporating a buffer window. We defined an anchor date at 7 days prior to the outcome date and extracted all the events from claims one year prior to the anchor date. A buffer window between the anchor date and the outcome date can help prevent information leakage from a methodological perspective and also allows physicians time to assess the potential risk and take action from a clinical perspective.

Regarding the study design of DL models to predict 30-day and 90-day hospital readmissions, the discharge date of the index HF hospitalization was defined as the anchor date (Figure 1). The data within one year prior to the anchor date were used to train the model. For traditional ML algorithms, we extracted the data from one year prior to the index date and used predefined covariates as input (Figure 1). Outcome labels were determined during the one-year period following the index date.

### Features

The features fed into the DL models included sociodemographic factors consisting of: age (18-64, ≥65); gender (male/female); geographic region (Northeast, North Central, South, West); insurance plan type (consumer driven health plan, comprehensive, exclusive provider organization, high deductible health plan, health maintenance organization, point of service with capitation, point of service without capitation, preferred provider organization, other plan, unknown); medical diseases and conditions; medical procedures; drug classes; and health-care resource utilization.

We also included the following pre-defined variables/features in the traditional ML models: 1) the sociodemographic factors listed above; 2) diseases and medical conditions observed including anemia, atrial fibrillation, chronic kidney disease including end stage renal disease, chronic obstructive pulmonary disease, asthma, coronary artery disease, depression, type 2 diabetes, hyperlipidemia, hypertension, myocardial infarction, peripheral artery disease, sleep apnea, stroke, thromboembolism, cancer, liver disease, and premature menopause; 3) medical procedures identified by procedure codes for cardiac resynchronization therapy, coronary artery bypass grafting, cardiac valve surgery, and percutaneous coronary intervention; and 4) health-care resource utilization (prior to the index date).

The DL models and traditional models adopted different approaches to process medical diagnoses (and conditions) and procedures features. The DL models vectorize diagnosis and procedure codes using the embedding matrix, which is a part of the model and specified in the next two sections. The traditional models utilized known hierarchical relations of medical codes to map individual diagnosis and procedure codes to the features listed in the previous paragraph.

### Deep Learning Model Design

**Figure 2.**
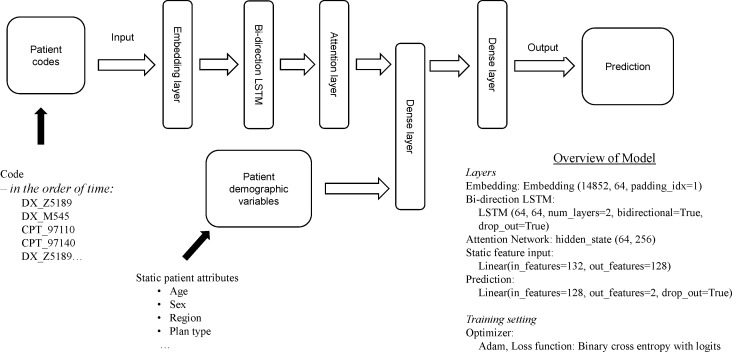
Deep Learning Model Design: Sequential Model Architecture Abbreviations: LSTM, long short-term memory.

Because of the naturally sequential format of longitudinal medical records, a sequential model architecture based on bi-directional LSTM (Bi-LSTM) layers^17^ was utilized as the DL model design (Figure 2). To represent the implied relationships between ICD-10 codes, all medical event codes (disease conditions, procedures, and medications) of our targeted patient population were grouped into sequences if the gap between two adjacent codes was no more than 7 days. The Bag-of-words model^18^ was used to create an embedding matrix^19^ based on all the short code sequences. This embedding matrix was used in our sequential model to convert the codes to numerical vectors, with the angle and distance between the vectors of any pair of codes in the latent space representing their relationship. We implemented vocabulary in PyTorch (ver. 1.5.1 with Torch Text 0.6) to assign corresponding embedding vectors for each medical code for each patient. The converted vectors were input into the Bi-LSTM layer with attention. LSTM networks provide good performance in sequential modeling tasks but are difficult to interpret. In addition, every element in a sequence is not equally important for predicting the final outcome. Moreover, as the length of the sequence increases, the model finds it challenging to focus on the most important elements within a sequence. The Attention mechanism^20^ addresses both challenges. Therefore, in this modeling pipeline, the output of the LSTM layer was fed into an Attention layer, which helped the model focus on the most important codes, ie, those that contributed to the binary outcome, and provided weights that indicate a measure of importance.

In addition to embedding the sequential claims data, we also performed feature engineering on sociodemographic characteristics and health-care resource utilization to include them as static variables in the modeling process. The attention layer’s weights on the codes were also concatenated with each patient’s demographic variables to make the final prediction using multiple dense layers.

For each outcome of interest, data were split into training (70%), validation (15%), and testing datasets (15%). The DL model was trained on the training dataset only, and the DL model with a specific parameter set that achieved the greatest area under the receiver operating characteristic curve (AUC) for the validation dataset was then re-evaluated against the testing data using the measurements of AUC, precision, and recall. The model was trained with the Adam optimizer.^21^ The loss function was binary cross entropy with logit loss (BCELogitLoss).^7^ Since the training dataset was imbalanced, the loss function was weighted with the positive weight being equal to the ratio of the majority class to the minority class. All the DL model training was conducted on Amazon’s SageMaker cloud machine-learning platform in Python 3.6 with a PyTorch 1.5.1 environment. Hyperparameter tuning was performed using training and validation datasets to optimize the learning rate of the Adam optimizer and the ratio of the two dropout functions in the Bi-LSTM layer and Prediction layer (Figure 2).

### Embedding Layer Training

In this study, we trained the embedding layer based on two different datasets: 1) the HFrEF patient cohort only, which only had 47 498 patients and more than 2 million medical events or encounters extracted from the claims (“small embedding”); and 2) a general patient cohort with all their data records (“big embedding”). During the embedding layer training process in both cases, the data used for training did not include the validation dataset, which prevented information leakage.

These different datasets determined the focus of the information learned by the embedding layer. The small embedding layer learned special information about HFrEF patients. The big embedding layer learned more general information from the general patient population but may be lacking in detail regarding HF patients. Both small and big embedding layers were used to train all DL models and compare their performance.

### Traditional Machine Learning Model Experiments

As comparisons, we tested multiple traditional ML models on the same patient group: logistic regression,^22^ Random Forest,^23^ and eXtreme Gradient Boosting (XGBoost).^24^ We adjusted class weights to fully offset the imbalance between the positive and negative classes. As described above, the study design is different from the DL modeling in that the independent variables regarding comorbidities, conditions, procedures, and health-care resource utilization were based on data from the one-year period prior to the index date, and all the outcomes were based on the data during the one-year period after the index date. For traditional models, 5-fold cross validation was applied to all available data, with average AUC, precision, and recall values on the validation portion reported in the Results section.

### Sensitivity Analyses

The Medicare portion of the MarketScan database contains only claims from patients with Medicare supplemental insurance. For these Medicare enrollees, we may not have complete medical information and records. To combat this issue, we conducted a sensitivity analysis of patients under 65 years old.

## RESULTS

There were 47 498 eligible HFrEF patients included in this study (Table 1). The majority of the patients were aged ≥65 years (62.3%), were male (60.0%), had preferred provider organization plans (40.8%), and lived in the South (37.3%). The average numbers of HF-related doctor visits and inpatient visits per month were 0.21 and 0.02, respectively. The rates of HF hospitalization and worsening HF events were 19.8% and 32.0%, respectively (data not shown in tables). Among those who had HF hospitalizations (n=9427), 9.1% of the patients had readmissions within 30 days following the HF hospitalization and 18.6% had 90-day readmissions (data not shown in tables).

**Table 1.** Characteristics of Patients with Heart Failure with Reduced Ejection Fraction (HFrEF)

**Sociodemographic Characteristics**	**N (%) (N=47 498)**
Age	
18 - 64	17 889 (37.7%)
≥ 65	29 609 (62.3%)
Gender	
Female	19 002 (40.0%)
Male	28 496 (60.0%)
Insurance Plan Type	
Consumer Driven Health Plan	2306 (4.8%)
Comprehensive	17 004 (35.8%)
Exclusive Provider Organization	285 (0.6%)
High Deductible Health Plans	1033 (2.2%)
Health Maintenance Organization	4199 (8.8%)
Point of Service without Capitation	2120 (4.5%)
Point of Service with Capitation	641 (1.4%)
Preferred Provider Organizations	19 356 (40.8%)
Unknown	554 (1.1%)
Geographic Region	
North Central	14 772 (31.1%)
Northeast	10 616 (22.4%)
South	17 697 (37.3%)
West	4371 (9.2%)
**Baseline Healthcare Utilization**	**Mean (SD)**
Number of All-cause Doctor Visits per Month	1.84 (1.51)
Number of HF-related Doctor Visits per Month	0.21 (0.37)
Number of HF-related Emergency Room Visits per Month	0.02 (0.06)
Number of All-cause Inpatient Visits per Month	0.05 (0.08)
Number of HF-related Inpatient Visits per Month	0.02 (0.05)

Abbreviations: HF, heart failure; SD, standard deviation.

### HF Hospitalization

The performance of the various DL and ML models in predicting HF hospitalization is presented in Table 2. Overall, DL models without a buffer window achieved much better performance (AUC 0.938-0.977) than those with a 7-day buffer window (AUC 0.492-0.595). Also, DL models using the small embedding layer performed slightly better than those using the big embedding layer, except for the total patient cohort and using a 7-day buffer window. Furthermore, DL models (with or without the buffer window) outperformed the traditional ML approaches among all adult patients with HFrEF (AUC 0.502-0.505).

**Table 2.** Model Performance in Predicting Heart Failure Hospitalization Among Patients with Heart Failure with Reduced Ejection Fraction

HF Hospitalization	AUC	Precision	Recall
Deep Learning Models (no buffer window)			
Using Small Embedding (Age ≥ 18)	0.977	0.913	0.974
Using Big Embedding (Age ≥ 18)	0.970	0.858	0.971
Using Small Embedding (18 ≤ Age < 65)	0.961	0.921	0.936
Using Big Embedding (18 ≤ Age < 65)	0.938	0.883	0.898
Deep Learning Models (7-day buffer window)			
Using Small Embedding (Age ≥ 18)	0.570	0.306	0.285
Using Big Embedding (Age ≥ 18)	0.573	0.303	0.312
Using Small Embedding (18 ≤ Age < 65)	0.595	0.290	0.426
Using Big Embedding (18 ≤ Age < 65)	0.492	0.175	0.429
Traditional Machine Learning Models (Age ≥ 18)			
Logistic Regression	0.505	0.442	0.014
Random Forest	0.504	0.510	0.009
XGBoost	0.502	0.534	0.005

Abbreviations: AUC, area under the receiver operating characteristic curve; HF, heart failure.

### Worsening HF Events

DL models without a buffer window achieved much better performance (AUC 0.943-0.972) than those with a 7-day buffer window (AUC 0.594-0.616) when predicting worsening HF events (Table 3). For the total adult sample, the best AUC achieved for DL models without a buffer window was 0.972 (using big embedding) and 0.608 with a 7-day buffer window (using big embedding), both of which were higher than the values attained by the traditional ML models (AUC 0.552-0.555). Only minor differences in AUC were observed between models using small embedding versus big embedding, as well as between models of the total adult sample and the subgroup of younger patients aged <65 years.

**Table 3.** Model Performance in Predicting Worsening Heart Failure Events Among Patients with Heart Failure with Reduced Ejection Fraction

Worsening HF Events	AUC	Precision	Recall
Deep Learning Models (no buffer window)			
Using Small Embedding (Age ≥ 18)	0.967	0.884	0.985
Using Big Embedding (Age ≥ 18)	0.972	0.903	0.984
Using Small Embedding (18 ≤ Age < 65)	0.962	0.907	0.961
Using Big Embedding (18 ≤ Age < 65)	0.943	0.861	0.943
Deep Learning Models (7-day buffer window)			
Using Small Embedding (Age ≥ 18)	0.594	0.438	0.563
Using Big Embedding (Age ≥ 18)	0.608	0.443	0.565
Using Small Embedding (18 ≤ Age < 65)	0.616	0.446	0.500
Using Big Embedding (18 ≤ Age < 65)	0.595	0.416	0.460
Traditional Machine Learning Models (Age ≥ 18)			
Logistic Regression	0.555	0.582	0.166
Random Forest	0.555	0.570	0.171
XGBoost	0.552	0.590	0.156

Abbreviations: AUC, area under the receiver operating characteristic curve; HF, heart failure.

### 30-day and 90-day Readmissions

The best AUCs for models of 30-day and 90-day readmissions following HF hospitalization using the DL approach in the total adult sample were 0.597 and 0.614, respectively, both of which were better than the values attained by the traditional ML approach (AUC 0.506-0.510 for 30-day readmission; 0.508-0.509 for 90-day readmission; Table 4). The DL model performance was substantially improved in predicting 90-day readmission when we restricted the patient age to <65 years (AUC 0.861 using small embedding and 0.837 using big embedding). Additionally, slight improvement in AUC was found in DL models using small embedding compared to those using big embedding.

**Table 4.** Model Performance in Predicting 30-day and 90-day Readmissions Among Patients with Heart Failure with Reduced Ejection Fraction

Outcomes	AUC	Precision	Recall
30-day Readmission Using Deep Learning Models			
Using Small Embedding (Age ≥ 18)	0.597	0.225	0.298
Using Big Embedding (Age ≥ 18)	0.568	0.270	0.188
Using Small Embedding (18 ≤ Age < 65)	0.557	0.250	0.313
Using Big Embedding (18 ≤ Age < 65)	0.488	0.154	0.145
30-day Readmission Using Traditional Machine Learning Models (Age ≥ 18)			
Logistic Regression	0.510	0.476	0.020
Random Forest	0.506	0.511	0.012
XGBoost	0.507	0.677	0.015
90-day Readmission Using Deep Learning Models			
Using Small Embedding (Age ≥ 18)	0.614	0.35	0.409
Using Big Embedding (Age ≥ 18)	0.581	0.331	0.329
Using Small Embedding (18 ≤ Age < 65)	0.861	0.652	0.928
Using Big Embedding (18 ≤ Age < 65)	0.837	0.634	0.885
90-day Readmission Using Traditional Machine Learning Models (Age ≥ 18)			
Logistic Regression	0.509	0.494	0.020
Random Forest	0.508	0.521	0.016
XGBoost	0.508	0.577	0.016

Abbreviations: AUC, area under the receiver operating characteristic curve.

## DISCUSSION

This study utilized a DL model based on LSTM to identify high-risk HFrEF patients using a large US nationwide commercial insurance dataset. For all outcomes assessed, the DL model outperformed traditional ML models. These results show the potential of DL to capture complex information from a massive dataset and set up a foundation for future research. In addition, the DL approach lowers the requirements for data formatting because the LSTM algorithm only needs the codes organized by time in order to place them in a sequence. By comparison, the traditional ML models require a manual feature engineering process, which may not be feasible or reliable in different clinical settings. This requirement limits the clinical utility of traditional ML models.

In predicting HF hospitalization and worsening HF events, DL models without buffer windows achieved the best model performance metrics, which were even better than the previously reported predictive models of HF hospitalization (C-statistic or AUC range: 0.590-0.800).^25^ However, using a 7-day buffer window between the anchor date and the outcome date, the models in this present study achieved AUCs of around 0.57 for HF hospitalizations and around 0.60 for worsening HF events. This buffer window is a novel element of our study design that, when incorporated, avoids information leakage and allows physicians time to identify the potential risk and take action, which is not common in previous studies using ML/DL algorithms to develop a predictive model.

For outcomes of 30-day and 90-day readmissions, our model performance is in the lower to middle range of previously reported predictive models of HF readmission (C-statistic or AUC range: 0.59-0.84),^3^ except for 90-day readmission in patients aged 18 to 64. There may be some plausible reasons for the relatively low AUC values for readmission outcomes in our study. First, medical and pharmacy claims data may not contain all the clinical information needed to predict health-care outcomes; most of the previously published studies utilized single-center electronic health record data, which may have more granular clinical data, yet may have limitations in terms of generalizability. This interpretation is consistent with a review of predictive models for HF hospitalization, which found that models attain better accuracy by using prospective electronic health record or registry data versus retrospective data.^2^ Another DL-based model using longitudinal electronic health record data achieved an AUC of 0.705 ± 0.015 in predicting 30-day readmission in HF patients.^26^ This difference could be due to the claims dataset missing key clinical information that reflects HF severity or progression. A recent review of ML applications in HF also found fair to poor performance when predicting 30-day readmission in HF and suggested potential limitations related to the lack of certain important data in the model, such as socioeconomic status and social support.^27^ Additionally, the sample size in this study may not be adequate for training complex DL models, ie, the number of positive outcomes may be low enough to cause an imbalance between positive and negative cases and therefore may substantially impact DL model performance.^28^ The high performance demonstrated in 90-day readmission in this subgroup may thus be due to the higher frequency of the outcome (29.3%). Even though we utilized regularization to alleviate this issue, it likely still had an impact on the model performance.

In terms of the comparisons between small and big embedding, the performance using the small embedding was generally equal to or better than that of using the big embedding. One plausible reason for this may be that the sample size and information in the current study cohort are adequate for training embedding. The large dataset, which contained information from all patients, may not add additional value to our DL models, instead just introducing more noise into them.

### Limitations

This study has several limitations. First, due to the nature of the dataset, we could not include some potential predictors such as lab results, biomarkers, social determinants of health, psychosocial factors, death, and personal health behaviors. Also, the study period may not reflect the most recent treatment patterns in HFrEF, which may need future investigation. Second, the sample size may be smaller than the DL model’s parameter size, which may induce overfitting. Third, the imbalance between positive and negative cases for outcomes especially for 30-day readmission following the HF hospitalization (9.1% positive cases vs. 90.9% negative cases) may have impacted the model performance. Fourth, the HF-related diagnoses and outcome measures were identified only using ICD-10 codes, which may possibly cause misclassification or underdiagnosis issues. Lastly, this dataset was derived from a subset of the commercially insured HFrEF population in the United States. And the inclusion criterion of a 12-month continuous enrollment after the index date may introduce a sampling bias of excluding the HFrEF patients with the highest risks. Thus, caution is needed when generalizing the model to the whole US HFrEF population. Nevertheless, this study can still serve as a foundation for further development and implementation of an innovative approach to analyzing other real-world data sources. In future research, more suitable regularization methods, models pre-trained on other datasets, and use of larger datasets with more granular clinical data are potential solutions.

## CONCLUSIONS

Our findings demonstrate the potential of DL using LSTM as a feasible and useful approach to predict HF-related outcomes. Further research is warranted to incorporate more clinical data to improve model performance. This study can help inform the future development and deployment of predictive tools in clinical practice, which can ultimately facilitate targeted strategies or interventions to improve outcomes in the HFrEF population.

### DISCLOSURE OF CONFLICTS OF INTEREST

Zhibo Wang, Xin Chen, and Mei Yang were employees of Merck Sharp & Dohme Corp., a subsidiary of Merck & Co., Inc., Kenilworth, NJ, USA, during the time of the study. Xi Tan, Lingfeng Yang, and Boshu Ru are employees of Merck Sharp & Dohme Corp., a subsidiary of Merck & Co., Inc., Kenilworth, NJ, USA, and stockholders of Merck & Co., Inc., Kenilworth, NJ, USA. Kartik Kannapur, Justin L. Vincent, and Garin Kessler are employees of Amazon Web Services Inc., which received research support from Merck & Co., Inc., to conduct the study.
